# Pathologic mallet fracture of distal phalanx enchondroma: A case report

**DOI:** 10.1097/MD.0000000000020219

**Published:** 2020-05-29

**Authors:** Byungsung Kim, Jae-Hwi Nho, Woo Jong Kim, Sungyong Park, Hak Soo Kim, Jahyung Kim, Hyoung Ye Kim, Ki Jin Jung

**Affiliations:** a Department of Orthopaedic Surgery, Soonchunhyang University Buchon Hospital, Bucheon; b Department of Orthopaedic Surgery, Soonchunhyang University Seoul Hospital, Seoul; c Department of Orthopaedic Surgery, Soonchunhyang University Cheoan Hospital; d Department of Orthopaedic Surgery, Seogwipo Medical Center, Seogwipo, Korea.

**Keywords:** distal phalanx, enchondroma, mallet fracture, pathologic fracture

## Abstract

**Introduction:**

Enchondromas arise from cartilaginous cells derived from the physis that persists throughout development. They are difficult to diagnose and are often discovered on radiographs after a trauma.

**Patient concerns:**

We discuss the case of a 32-year-old woman with a pathologic mallet fracture of the distal phalanx that was initially misdiagnosed as common mallet fracture.

**Diagnosis:**

Magnetic resonance imaging revealed an eccentrically located lesion expanding to the cortex, with a high signal intensity at T2 weighted image, suggesting pathologic fracture.

**Interventions:**

We performed a operation, involving curettage and bone grafting. Complete removal of the lesion was confirmed intraoperatively with the image intensifier, and the cavity was irrigated and subsequently filled with allogenous bone and demineralized bone matrix.

**Outcomes:**

With an uneventful recovery, she gradually return to normal function within 3 months. Her symptoms improved and nearly full range of motion of the finger was seen at 1-year follow-up.

**Conclusion:**

Our case of pathologic mallet fracture was misdiagnosed as a simple fracture, suggesting that the mechanism of fracture and radiological diagnosis should be carefully considered.

## 1 Introduction

Enchondromas are the most common and destructive primary bone tumors of the hand.^[1]^ They arise from a nidus of cartilaginous cells derived from the physis that persists throughout development.^[2]^ The tumor arises in the medullary cavity and grows outward into the cortex, forming a prominent endogenous mass in the bone.^[2]^ Because enchondromas have no unique clinical symptoms, they are difficult to diagnose and often remain undiscovered until patients require radiographs after a trauma. Pathologic fracture is a common complication because minimal trauma can fracture the thin shell of bone. Therefore, physicians need to be cautious when taking patient history, conducting physical examination, and interpreting radiographic images. The most frequent location of enchondromas is the proximal phalanx, followed by the middle phalanx and the metacarpals, with rare occurrence in the distal phalanx.^[3]^ We discuss the case of a 32-year-old woman who had a pathologic mallet fracture of the distal phalanx that was initially misdiagnosed as common mallet fracture.

## 2 Case report

A 32-year-old right-hand dominant female visited our orthopedic clinic with a history of acute-onset painful swelling of her right 4th finger. On examination, there was diffuse swelling of the distal phalanx of the 4th finger with extension lag at the distal interphalangeal (DIP) joint. The patient reported that her finger was sprained when taking her clothes off.

**Figure 1:**
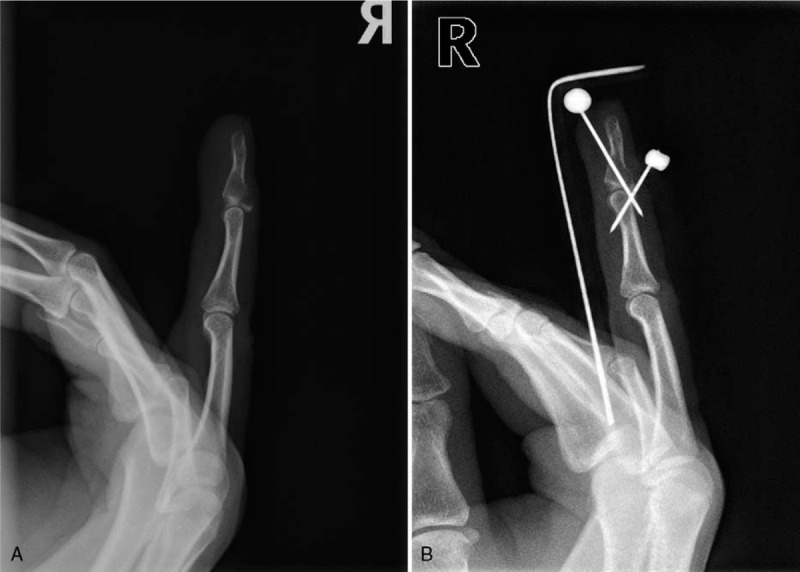
(A) Initial lateral plain radiographs of the patient's hand (B) Postoperative radiographs following extension block pinning with Kirschner wires.

**Figure 2:**
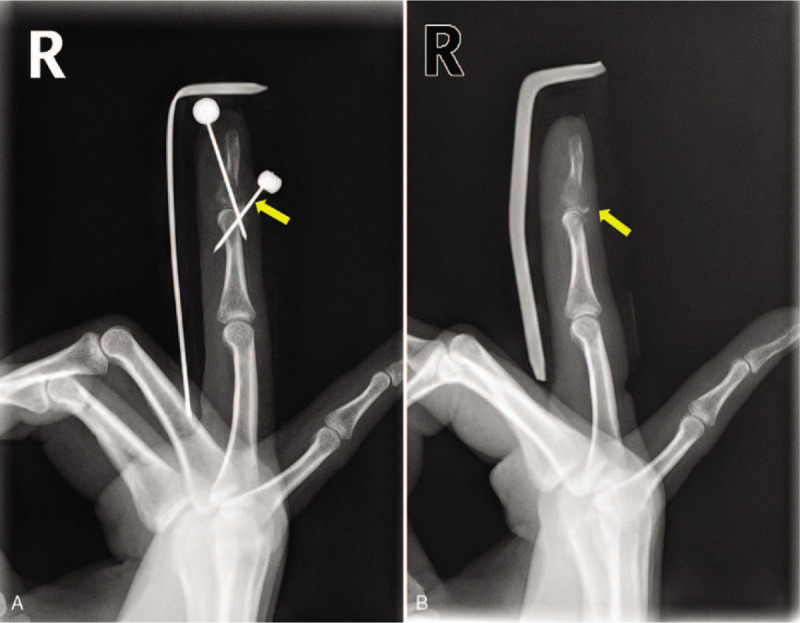
Osteolytic lesion seen on 2-wk postoperative radiographs.

**Figure 3:**
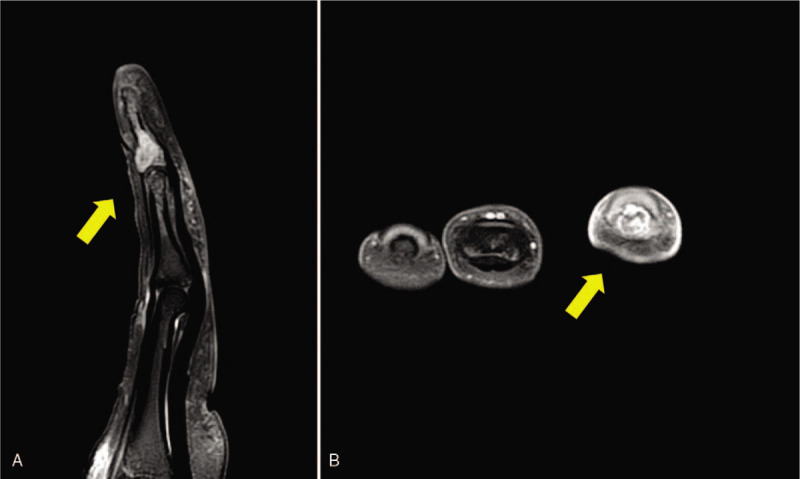
Magnetic resonance imaging demonstrating an eccentrically located mass that had expanded to the cortical bone.

Initial clinical and radiologic examinations showed a mallet fracture in the 4th finger of her right hand (Fig. 1), and closed reduction and extension block pinning with Kirschner wire was planned. The involvement of the joint surface was less than 30 percent, but the fragment had been displaced and the operation was decided. Surgery was performed under local anesthesia with a digital block. Extension block pinning was performed using Kirschner wire, and we expected her finger to be united (Fig. 1). Two weeks postoperatively, however, an osteolytic lesion was evident on the plain radiograph and turbid fluid discharged from the pin site, leading to a diagnosis of post-operative infection (Fig. 2). Intravenous antibiotics were initiated and the pins were removed from her finger for magnetic resonance imaging, which revealed an eccentrically located mass-like lesion that had expanded to the cortex and that had a high signal intensity on T2-weighted imaging, suggesting pathologic fracture (Fig. 3).

**Figure 4:**
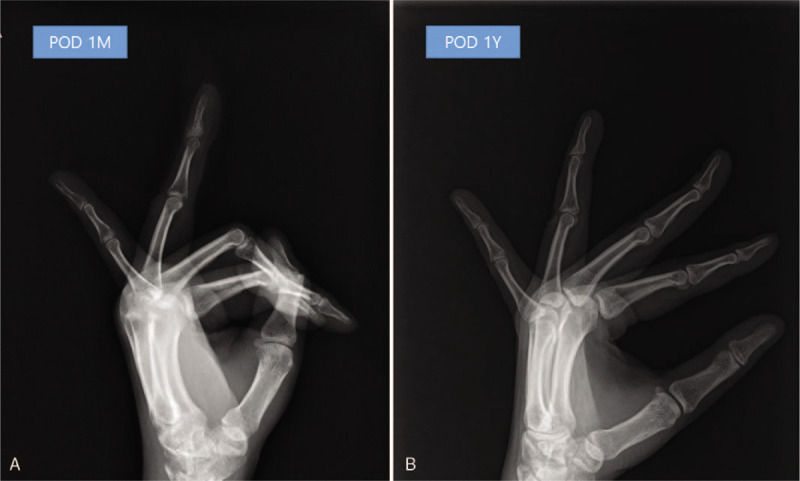
Radiographs showing postoperative results of curettage and bone grafting at 1 mo (left panel) and 1 yr (right panel). Full union of the pathologic fracture was evident at 1 yr postoperatively.

Serial review of plain radiographs revealed an enchondroma located in the distal phalanx of 4th finger (Fig. 1). Curettage and bone grafting were carried out under local anesthesia with digital block and tourniquet control. A longitudinal incision at the dorsal aspect of the distal phalanx was made and a small window was created at the tip of the distal phalanx for insertion of a micro curette under C-arm image control to improve access and allow cautionary approach to thin areas of the cortex. The lesion was removed piecemeal and complete removal was confirmed intra-operatively. The cavity was irrigated and subsequently filled with allogenous bone and demineralized bone matrix. The access incision was sutured with 5/o nylon, and the finger was splinted. The patient made an uneventful recovery with gradual return of function in 3 months (Fig. 4). Her symptoms improved and she had a nearly full range of motion in her finger at the 1-year follow-up. Informed written consent was obtained from the patient for publication of this case report and accompanying images.

This case report was approved by the International Review Board of Soonchunhyang University Hospital.

## 3 Discussion

Enchondroma accounts for approximately 90% of hand tumors.^[3]^ They frequently develop in the proximal phalanx.^[4]^ Distal phalangeal enchondromas are rare and often difficult to differentiate from epidermal cysts, glomus tumor and osteoid osteoma.^[3]^ Clinical feature of these lesions are variable, the glomus tumor present changes in pain severity depending on temperature. The epidermoid cyst causes more deformity of the nail than the glomus tumor and enchondroma. Both tumors involve more aggressive clinical feature than enchondroma and they frequently present before causing a pathologic fracture.^[4]^

Enchondroma of the hand has a nonspecific clinical presentation and a variable radiographic appearance.^[4]^ Although many cases of enchondroma are discovered incidentally, the most common presenting signs and symptoms are nonspecific and include pain, swelling, and deformity. Fractures may arise during seemingly trivial activities, such as moving or carrying objects.^[5]^ On rare occasions, active DIP joint flexion may be lost secondary to an avulsion injury of the flexor digitorum profundus tendon from the underlying, weakened volar cortex.^[6]^ However, this patient presented a sudden DIP joint flexion with finger extension, resulting in immediate functional disability. We missed this pathologic fracture because we overlooked that this fracture was due to trivial activities. Many pathologic fractures occur after low-energy injuries. When pathologic fracture of the hand due to enchondroma is diagnosed, although there are no clear guidelines for the treatment. many hand surgeons delay surgery until union of the pathologic fracture has been achieved.^[7]^ Traditionally, enchondroma-related pathologic fractures have been treated with 1 to 2 months of immobilization to allow healing before curettage.^[8]^ The decision to delay treatment is controversial, however, and more recent reports address immediate, 1-stage treatment of both nondisplaced and displaced fractures.^[9]^

We report a case of pathologic mallet fracture that was misdiagnosed as a simple fracture, indicating that the mechanism of fracture and radiological diagnosis should be considered more precisely.

## Acknowledgments

The authors would like to thank the Soonchunhyang University Research Fund for support.

## Author contributions

**Investigation:** Woo Jong Kim.

**Resources:** Sungyong Park, Jahyung Kim.

**Software:** Hak Soo Kim, Hyoung Ye Kim.

**Writing – original draft:** Byungsung Kim, Jae-Hwi Nho.

**Writing – review & editing:** Kijin Jung.
